# Commentary: Disulfidptosis-related gene signatures as prognostic biomarkers and predictors of immunotherapy response in HNSCC

**DOI:** 10.3389/fimmu.2025.1575090

**Published:** 2025-03-25

**Authors:** Jihao Xue, Cheng Xue, Qijia Yin, Ligang Chen, Ming Wang

**Affiliations:** ^1^ Department of Neurosurgery, the Affiliated Hospital, Southwest Medical University, Luzhou, Sichuan, China; ^2^ First Affiliated Hospital of Nanchang University, Nanchang, Jiangxi, China; ^3^ Department of Center for Renal Diseases, Sichuan Provincial People’s Hospital East Sichuan Hospital & Dazhou First People’s Hospital, Dazhou, Sichuan, China

**Keywords:** half maximal inhibitory concentration (IC50), medication, disulfidptosis-related genes (DRGs), head and neck squamous cell carcinoma (HNSCC), immunotherapy

**Figure 11:**
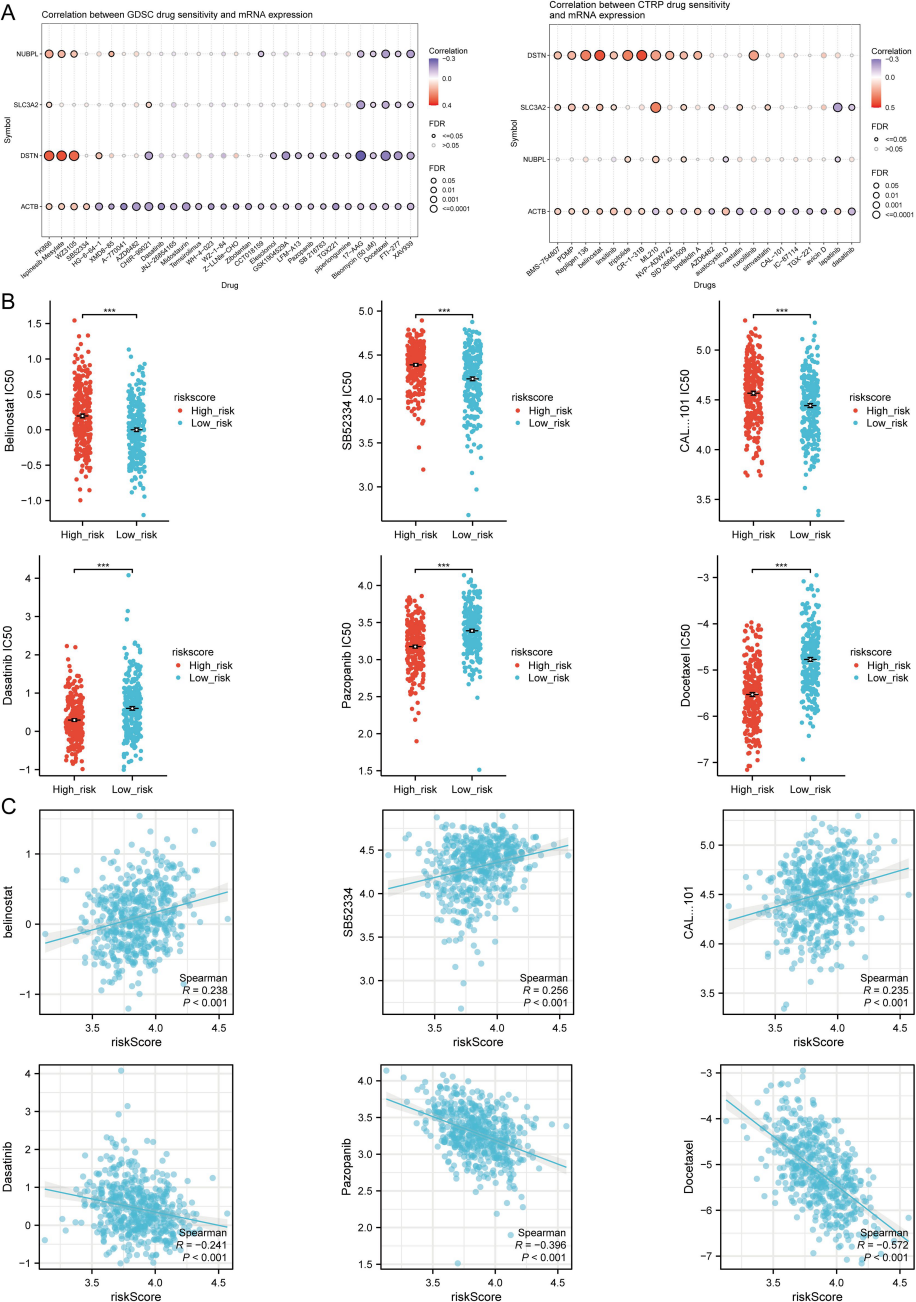
Drug sensitivity analysis. **(A)** Predictive antitumor drugs based on the three prognostic DRGs expression in HNSCC from the GDSC and CTRP datasets. **(B)** The distribution of IC50 scores in the high and low risk groups. **(C)** Spearson correlation analysis of IC50 score and riskscore. ***p<0.001. (Image source: Qin et al., Disulfidptosis-related gene signatures as prognostic biomarkers and predictors of immunotherapy response in HNSCC).

We read with great interest the research “Disulfidptosis-related gene signatures as prognostic biomarkers and predictors of immunotherapy response in HNSCC” by Qin et al. (1), which was recently published on Jan 17, 2025, in the Journal of *Frontiers in Immunology*. This article elucidated the potential significance of disulfidptosis-related genes (DRGs) in head and neck squamous cell carcinoma (HNSCC), as demonstrated through rigorous bioinformatics analysis and experimental validation. Through in-depth mechanistic investigations, they revealed the mechanisms by which these genes regulate tumor cell death and influence the functionality of immune cells within the tumor microenvironment. Additionally, the efficacy of these genes in forecasting patients’ responsiveness to immune checkpoint inhibitors was assessed, offering valuable insights for the advancement of novel therapeutic strategies. Although acknowledging the significant contribution of that study, Qin et al. had misunderstood **Figure 11B** in the section titled “TMB, MSI, mRNAsi, and Drug Sensitivity Analysis”—they believed that in high-risk HNSCC, the sensitivity of belinostat, SB52334, and CAL101 was significantly higher than in the low-risk group, while Dasatinib, Pazopanib, and Docetaxel showed higher sensitivity in low-risk HNSCC (1).

IC50, defined as the half maximal inhibitory concentration, signifies the concentration at which a drug or inhibitor diminishes the activity of a biological process (such as an enzyme, receptor, or cell) to half of its maximum level under specific experimental conditions. A lower IC50 value indicates that the drug can achieve a 50% inhibitory effect at a lower concentration, suggesting a higher potency and sensitivity of the drug (2, 3). Therefore, the correct interpretation of **Figure 11B** in the original text of Qin et al. (1) is that in HNSCC patients, the IC50 values for belinostat, SB52334, and CAL-101 are significantly higher in the high-risk group compared to the low-risk group, suggesting lower sensitivity of the high-risk group to these drugs. Conversely, the IC50 values for Dasatinib, Pazopanib, and Docetaxel are significantly lower in the high-risk group, indicative of higher sensitivity of this group to these drugs relative to the low-risk group.

In summary, readers should exercise caution when reading the appropriate chapters to ensure accurate comprehension.
